# Home Correspondence

**Published:** 1871-09

**Authors:** 


### Home Correspondence.

CHICAGO, AUG. 3, 1871.

*Dear Professor:*

Your aim is to "elevate the profession" by occasionally "blowing it up," and it seems to me, as well as to many of your readers, that your aim is good and effectual. You and I have jogged along through life together in our great work for over a quarter of a century, and ever since you assumed charge of the JOURNAL have I been an earnest reader of it, and been blown up by you repeatedly. I have been benefited greatly by your effervescent editorials ; but now, as a slight return, will you allow *me* to "elevate" a trifle? Can you submit gracefully? *Nous verrons.*

You love old things—old ways—so do I; you are chary of innovations—so am I. Any one advancing a new-fangled notion must substantiate it or be the butt of your ridicule and mine. Female suffrage and woman doctors are questions that awaken your profane proclivities and silent contempt, and mine, also. Did you notice the arguments on the question of countenancing women doctors, in the late National Medical Association in California? We can hardly withhold our dissent from their conclusions—but it seems to me that the majority of the doctors bubbling over with indignation against the women are narrow-visioned—decidedly rutty. Do we men contain all the wisdom and ability in creation? This question is one of the period and must be decided by us, or else public opinion will decide for us, and thrust the decision down our throats, and laugh at our wrigglings and wry faces. We might much better use the philosophy of the old judge we are told about in the Bible—"If this comes from God, man cannot overthrow it; if *not*, it will die of itself." Opposition will surely develop every atom of strength of every question, on which the public split, and hearty coincidence will usually kill any nonsensical scheme.

But this is not what I started out to say. Why are we so mortally opposed to Homœopathy? What is there in the idea of Similia that makes us bristle up and announce our readiness to masticate some small-pill man, when the new doctrine is mentioned in our presence? In ordinary affairs of life we smile at a man who works himself up into a rage over an *imaginary* evil. What is *our* strong argument against small-pillism? *always*, that it is impossible to get results from such small amounts of medicine. From this basis we satisfactorily conclude that all practice of the infinitesimal school is indebted for its success to the curative powers or tendencies of nature. The amount of water required to raise a dilution of medicine to the 30th rank is simply astounding. Mind cannot conceive such a vast sphere of liquid, and when we narrate to our patrons, or any one else seeking information on the point, we are greeted with satisfactory evidence of the pungency of our argument, from our respectful listeners. There are many things that make us think about this subject, and sometimes we become most amiably savage before we turn our thoughts into another channel. You and I have both of us lost some very estimable families by the new doctrine. At first we chuckled over their prospective chagrin and disgust, and at no distant day certain return to us. Many, very many, *did* return—but the balance *stuck.* When the latter were our patrons, we thought them tolerably clever. Now—ah! well they are deluded, to put it mildly. It has been cheering, also, to note the converse, and see how many patrons who have *always* been favorable to the new doctrine, abandon it and come over to us.

You and I are fast passing into the sere leaf, and new ideas are not going to worry us much. We practice as we think proper, and our medicines yield us good results, and we care not to turn over many more leaves. Yet we acknowledge there are new modes of cure daily coming to light in our school—every shade and variety of opinion on medicines are being put forth, and good results come from them all, and we take note of them with genuine satisfaction. We grow daily: and what more is needed to complete our happiness in our declining days, when we see such great minds in our profession as greet us at every turn, using their utmost endeavors to advance and perfect our science? We abominate fogies, and stand aloof from radicalism; we belong to neither, and are willing to hurt any one who accuses us of being classed with either category. Hence it is we laugh at Prof. P. of Michigan University, for his determination to have every man bled in pneumonia and pleurisy (and at the same time launch forth his annual bull of excommunication against Jno. Hughes Bennett who thinks otherwise), and at another professor of Theory and Practice who fathers the American Med. Assoc., for his prescriptions containing so many and such incompatible remedies, and at the same time hold up our hands in holy horror at the course taken by the Albany Med. College in electing a professor to represent Homœopathic practice! The Leopoldstadt Hospital of Vienna has an Allopathic section and a Homœopathic section. Both schools are nurtured and cared for by the government. The Surgeon General of Bavaria is a Homœopath.

The question naturally comes to us, "Is there anything in Homœopathy worth learning?" Has that school any one remedy or mode of treatment of one disease even superior to ours? If it has, why not adopt it? A "Regular," in administering a remedy, never inquires "Is this medicine to act by absorption—revulsion—catalysis—mechanically—chemically, or through the influence of the mind on the body?" He gives it because he knows its effects, and is assured it will do what he wants it to do. Take castor oil for instance—it catharticises. *How* does it do that? By its action on the bowels. Precisely; but *what* is that action? Most medicines we know the action of, but *how* they act is no easy matter to explain. Simply because this artistic class of physicians announce with trumpet flourishings, that all diseases must be cured on the "Similia" principle, do we thrust their teachings from us and listen to none of their precepts. If they have ever so many grains of truth we deprive ourselves of them simply because they come from this source. Is that wisdom? Perhaps some junior practitioner, having read this article thus far, is thoroughly disgusted with it, considers the writer a doting old ass for consenting to even talk about such elegant nonsense as Homœopathy. It makes me smile to think even that; yet you and I know that such a young man has much to unlearn and much to learn. We know that at first sight it would seem to be an easy matter to settle beyond a doubt the efficacy of medicines. Hence, so many young men, starting out every year, confident of their medicines accomplishing precise results foreknown, soon come to grief, by being lost in their "doubting stage." They soon learn, that although diseases follow such and such courses after administering certain remedies, it, unfortunately, becomes one of the most difficult problems in practical medicine, to appreciate correctly the value of any plan of medication. You and I have done some very good service with small doses of calomel and ipecac. With the former we *check* a bilious diarrhœa—and with the latter we *check* obstinate vomiting. Yet every one knows that large doses of calomel will produce bilious diarrhœa, and large doses of ipecac will produce vomiting. That is good Homœopathy, too—such cures. *How* do those remedies act? Ringer's new Materia Medica gives many properties of different drugs—*e. g.*, camphor, ipecac, aconite, arsenic, iron, etc.,—which hitherto have been found only in works on Homœopathy. If our principles of medication and treatment of disease are *good*, why are we continually changing? Every work on Practice and Materia Medica exhibits the want of fixed, stable principles in our therapeutics. Twenty-five years ago we gave large doses of everything, including even calomel—and bleeding was extraordinarily common—as the scars on your arm and mine will attest. Then we were tinctured with the idea that we must head off or neutralize or kill the disease—"cut short the fever"—and to-day we are laboring in the majority of cases, as in labor, to conduct them safely to the end.

In books and lectures nowadays we are taught that we are *physicians*,—not "regulars," "allopathic," "old school," or any other kind, but simply physicians. We recognize no distinctions—we want no adjections to qualify us—no prefixes. We propose to use *every*thing and *any*thing to cure diseases, be it medicines, condensed or rarified air, heat, cold, water, electricity, health lift, or anything else; but when we come to cure diarrhœa with camphor in drop doses repeated often, or croup with bryonia and aconite in frequent installments, we are at once becoming quackish, solely because the homœopathic school uses it. What do we care for the "homœopathicity" of camphor or bryonia or any medicine so long as it serves our end and furnishes us with a uniform remedy? What we want is truth, and using such a remedy cannot weaken us, nor does it entail our sanctioning such nonsensical ideas as the 30th of pulsatilla turning the fœtus in utero. My attention has been called frequently of late years to this subject by a brother and sister who use homœopathic treatment in their families. Having been candid with myself, and convinced that small pills have *accomplished cures* in their households, I am not talking blindly, but know whereof I affirm. I have seen cholera infantum checked successfully by this system of medication, and diarrhœas of young nieces and nephews ad infinitum, almost, cured by the same method. If Homœopathy does good in families, it is almost invariably accompanied by one reprehensible bad habit, viz: the incessant inclination to magnify every trifling "symptom," and pour down the pellets and powders. In every well regulated "homœopathic" family of—say three or four children, hardly a day, never a week, passes without a resort to superfluous medicine. Four-year-old Charlie, a delicate boy, plays pretty hard all day, and at the supper table finds he is too tired to eat, and perhaps has a trifling fever in addition. Here we have two grave "symptoms." Mamma looks very wise, papa interested, and directly down goes a dose of aconite or belladonna, to be followed by another in thirty minutes. Charlie is put to bed and anxiously watched over; faces are solemn enough and surmises dreadful. Sonny is surely going to have typhoid fever, chicken pox, or scarlet fever. A good night's rest finds the patient rested, hungry, bright, and ready for another day's romping. Friends are duly informed of the wonderfully mysterious powers of potencies and pellets in arresting typhoid fevers and scarlatina, and up goes the united chorus from all, "Great is Homœopathy!"

Putting aside all foibles and evil tendencies of this peculiar school, why is it not incumbent on us to sift the wheat from the chaff and grind it for our own benefit? Homœopathy's worst enemies are its own advocates. The chief reason of its unpopularity with the scientific world at large, is the extravagant and outrageous claims put forth by its adherents, too large a proportion of whom are scarcely fit to act as a doctor's coachman. Occasionally we meet with an educated doctor of this school who is *posted* in medicine and its allied sciences, who reads our journal and his, who, to secure *ends* uses *means*, whether condensed or attenuated, large or small, quarter grain doses of morphia, fifteen-grain doses of chloral or the thirtieth of belladonna to procure sleep if necessary, yet he adheres to the Similia doctrine in most points, claiming that Homœopathy is undeveloped, and that at some day he can get along without resorting to large doses! Most doctors of this ilk, he affirms, are like a man journeying to a far country, by rail and coach, determined to use rail only, and when a gap of the former communication occurs refuses to use the latter; whereas he uses both means, comforting himself with the assurance that some day all will be a uniform means of transit.

Now then, sir, cannot you, as a teacher of young doctors, and Doctor Ingals, do great service to the profession by encouraging young men to pick up all good points connected with Homœopathy and adopt them, so far as they are sure and efficatious? Drop-doses of tincture of aconite given hourly "break up a cold;" vapor bath will do the same; Turkish bath will do the same; acetate of potash, spirits nitre, serpentaria, an hour or two of violent exercise, a bottle of citrate of magnesia, sometimes a full opiate at bedtime, or larger draughts of water at night, will all accomplish the same end. Do you, in your annual course of lectures, advocate confining treatment of a given disease to any *one* line of medication? Certainly not. Frequently you tell the young men of as many different means of cure of a disease as I have enumerated for a "cold." A young man well balanced is not going to be contaminated, or run off into any special ism, by reading homœopathic books. Isn't it wisdom to encourage free reading of all medical doctrines? Young men turned out of our colleges now-a-days would as soon be found reading a homœopathic book as a Catholic would be found reading a Protestant bible. I have been often greatly amused by brother doctors looking over my library and noticing several standard homœopathic works therein, and expressing astonishment thereat.

The history of our profession records the birth and death of many new doctrines. Each doctrine had its adherents and enemies, who fought, bled and died over it, and it always left the profession, upon its demise, in better "shape" than it found it at birth. Just so Homœopathy has taught us two things:—1. That sick people will recover with small amounts of medicine or without any at all. 2. That we can devise means of administering nauseous compounds devoid of repulsiveness. The first item has almost totally revolutionized our therapeusis, and the second has called forth many ingenious and pleasant means of conveying anything, almost, into the most delicate systems.

If you publish this communication, my dear Professor, I expect a huge return of invective and ridiculous abuse from my veriest friends. Can you afford to put it in your JOURNAL?

Affectionately, Votrè Frere, SENEX.

[The senior editor departs from his usual custom in admitting the above communication, without the guarantee of personal knowledge of the author's name. The advance of medical science, and improvement of medical practice, which our anonymous friend refers to Homœopathy, we refer to the advance of physiological discovery. Homœopathy, at the best, is but one form of morbid anatomy. But morbid anatomy, rightly considered, is one of the most important subordinate studies of the Physiologist. "There is a soul of goodness in things evil."—ED.]

